# 40-Hz ASSR fusion classification system for observing sleep patterns

**DOI:** 10.1186/s13637-014-0021-2

**Published:** 2015-02-05

**Authors:** Gulzar A Khuwaja, Sahar Javaher Haghighi, Dimitrios Hatzinakos

**Affiliations:** Department of Electrical and Computer Engineering, University of Toronto, 40 St. George Street, Toronto, ON M5S 2E4 Canada

**Keywords:** Adaptive classification, Observing sleep patterns, Features-level fusion, ASSR extraction, Depth of general anesthesia (DGA)

## Abstract

This paper presents a fusion-based neural network (NN) classification algorithm for 40-Hz auditory steady state response (ASSR) ensemble averaged signals which were recorded from eight human subjects for observing sleep patterns (wakefulness *W*_*0*_ and deep sleep *N*_*3*_ or slow wave sleep *SWS*). In *SWS*, sensitivity to pain is the lowest relative to other sleep stages and arousal needs stronger stimuli. 40-Hz ASSR signals were extracted by averaging over 900 sweeps on a 30-s window. Signals generated during *N*_*3*_ deep sleep state show similarities to those produced when general anesthesia is given to patients during clinical surgery. Our experimental results show that the automatic classification system used identifies sleep states with an accuracy rate of 100% when the training and test signals come from the same subjects while its accuracy is reduced to 97.6%, on average, when signals are used from different training and test subjects. Our results may lead to future classification of consciousness and wakefulness of patients with 40-Hz ASSR for observing the depth and effects of general anesthesia (DGA).

## Introduction

The manual scoring of sleep patterns is a time-consuming process, consisting of the determination of sleep states using an electroencephalograph (EEG) signal. Automatic classification has been studied in sleep scoring extensively [1-3] and is considered an important tool in biomedical research. Although good results have been achieved using EEG, the classification of human EEG signals continues to be a difficult problem due to the high-dimensional and noisy nature of EEG data [4].

Auditory steady state response (ASSR) is a brain auditory evoked potential (AEP) produced with a periodic stimuli with a 40-Hz repetition rate. AEP is produced as a result of electrical changes in the ear and brain of a normally hearing person in response to acoustic stimuli. An AEP signal shows how neural information propagates from the acoustic nerves in the ear to the cortex [5]. Specifically, AEP signals are extracted from EEG [6,7]. The auditory stimuli are either the repeated clicks or tone bursts that vary in frequency and rise time. If the stimulus lasts long enough to get the response to its steady state, then the signal is called ASSR [6]. AEP and ASSR signals are mainly used as audiology tools for predicting the hearing threshold and sensitivity of an individual.

**Figure 1:**
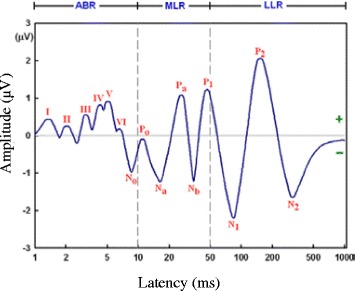
**Auditory evoked potential.**

In 1950, the first clear approach to distinguish the evoked response from background EEG was made by Dawson [8]. First AEPs were generated by averaging the EEG response by Geisler et al. [9] in 1958. Later in 1980s, the 40-Hz ASSR was described by Galambos et al. [10]. An ASSR signal is called a 40-Hz response when the stimulus has a repetition rate of around 40 Hz. The amplitude of AEP signal is much smaller than the amplitude of EEG signal, hence extracting the AEP from the background EEG is a challenging process that involves noise cancelation techniques. AEP is divided into three main parts, namely, auditory brain stem response (ABR), mid-latency AEP (MLAEP), and late latency AEP (LLAEP) [5,11-13]. Figure 1 shows an AEP signal.

The ASSR is greatly affected by the stimuli modulation rate and is phase locked and follows the modulated envelope of the stimulus [14]. Different stimulus rates result in stimulation of different portions of the auditory nerves and hence produce different ASSRs. Specifically, ASSRs stimulated with stimuli of lower than 20 Hz reflect the activity of LLAEP generators. ASSRs stimulated with stimuli of 20 to 60 Hz are generated by the same generators as MLAEP generators, while those stimulated with stimuli of above 60 Hz are generated by ABR generators [14,15].

Galambos et al. [10] demonstrated that when stimuli presented at the rates of 30 to 50 Hz, the amplitude of the response was two to three times greater than the amplitude of the transient MLAEP in response to stimulus presented at 10 Hz. A 40-Hz response, like that produced by MLAEP has small inter- and intra-subjective variations [5,16], but it is strongly influenced by the subject's state of arousal [8]. Correspondingly, the amplitude in a 40-Hz response varies by the subject's level of arousal [10,14,17,18] and consciousness [19,20]. A 40-Hz response can be used as a measure of depth of general anesthesia [20-24].

Preliminary classification results of this research work have been published using linear discriminant analysis (LDA) and quadratic discriminant analysis (QDA) classifiers [25]. In this work, the average error rate for training and testing with the same subject is 1.12% with LDA and 1.66% with QDA. LDA has an acceptable error rate of 2.57% but the QDA error rate increases to 17.43% for six subjects. In other situations, the classifiers are trained with ASSRs from all subjects except for the subject whose ASSRs is to be classified. The average error rate over all subjects in this case is 5.91% with LDA and 20.69% with QDA.

Neural networks (NNs) are fundamentally analog, non-programmed data processing structures [26]. The networks are comprised of processing elements, each of which has a set of inputs, a set of weights, and one output. Inputs are multiplied by their weights and summed. The output is computed as a non-linear function of the summation. They offer fine-grained parallelism and exhibit fault tolerance. One advantage of any NN, which performs a classification task, is that it will learn its own coarse-grained features, thus does not require precise locations to form any part of an input set [27].

Classification of signal patterns is the most common NN applications. It has been demonstrated with artificial as well as natural data [28-32] that the learning vector quantization (LVQ) methods [31-34] constitute a very viable alternative to the more traditional classification approaches. LVQ classification accuracy is as good as other NN algorithms or better, whereas because of the very simple computations are applied, the learning and classification speed can be considerably higher as compared to other NN algorithms [35]. Also, LVQ methods are very easy to use.

Additionally, support vector machine (SVM) classification is incorporated. SVMs are supervised learning models with associated learning algorithms that analyze data and recognize patterns used for classification and regression analysis. Given a set of training examples, each marked as belonging to one of two categories, an SVM training algorithm builds a model that assigns new examples into one category or the other, making it a non-probabilistic binary linear classifier.

This research focuses on a fusion-based NN system classification of 40-Hz ASSR signals recorded from eight subjects used in observing sleep patterns in humans. The purpose of this work is threefold: a) to generate an automatic classification of sleep patterns (wakefulness *W*_*0*_ and deep sleep *N*_*3*_) based on an adaptive LVQ-NN and SVM with 40-Hz ASSR input signals, b) to develop a features-level fusion approach for combining a 40-Hz ASSR ensemble averaged sweep signals generated from two separate electrodes/channels, and c) to classify sleep patterns with the resultant ASSR sweep signals to enhance the decision confidence level.

The remainder of this paper is organized as follows: a view of previous related work is given in Section 2. Section 3 describes various stages of database acquisition from eight human subjects during sleep cycles. This is followed by a description of data extraction in the form of an ASSR ensemble of averaged sweep signals from EEG generated for classification. In Section 4, the overview of the algorithm including the proposed features-level fusion approach based on an adaptive LVQ-NN architecture is presented. The empirical results are discussed in Section 5. Finally, in Section 6, the paper is concluded.

## Previous work

The most common physiological signal used for sleep discrimination in clinical settings is the recording of brain activity with an EEG [36]. One of the important uses of observing sleep patterns of subjects at home is early detection of sleep disorders resulting in prompt intervention and reduced health care costs [37]. In 1986, Jarger et al. [18] studied ten subjects in three stages: awake, stage 1, and stage 2 of sleep. The 40-Hz ASSR signal was generated by averaging over 128 sweeps. Amplitude and phase of the 40-Hz component of the fast Fourier transform of the signal was considered. They observed that while sleeping affects the amplitude, phase coherency remains unaffected by the level of subject arousal. A fuzzy logic approach to the classification of human sleep using EEG data is presented in [38]. In this approach, frequency and amplitude information from an epoch of the EEG signal are extracted into a vector that is then compared to previously taught vectors representing the canonical features of six stages: wakefulness, rapid eye movement (REM) sleep, and four non-REM sleep stages. For each stage, membership functions are calculated in each epoch. The stage with the maximum degree of membership is scored and classified. The system is implemented in software using the C programming language. Analysis of about 1,101 epochs of the EEG data yielded an overall agreement of 77% between the program and a human scorer.

Suzuki et al. [39] recorded 40-Hz ASSR signals from 12 subjects with normal hearing in awake and stage 2 of sleep. They compared the 40-Hz recorded SSR with synthesized SSR signals generated from superimposing the recorded ABR and middle latency response (MLR) signals. Required signal-to-noise ratio (SNR) reduction for the 40-Hz ASSR signals was achieved by averaging over 2,048 sweeps. They observed that the amplitude of the 40-Hz ASSR signal in awake state is twice as large as in sleep state and that the synthesized 40-Hz ASSR cannot predict accurately this reduction in amplitude. Lewicke et al. [40] reliably determined sleep and wake states using only the electrocardiogram (ECG) of infants. The method was tested with simultaneous 8-h ECG and polysomnogram (PSG) determined sleep scores from 190 infants enrolled in the collaborative home infant monitoring evaluation (CHIME) study. LVQ neural network, multilayer perceptron (MLP) neural network, and SVMs were tested as the classifiers. After systematic rejection of difficult-to-classify segments, the models could achieve 85% to 87% correct classification while rejecting only 30% of the data.

To overcome the limitations of inter-subject variability, Kalrken and Floreano [41] suggested a novel online adaptation technique that updates the sleep/wake classifier in real time and evaluated the performance of a newly developed adaptive classification algorithm that was embedded on a wearable sleep/wake classification system called SleePic. Their proposed algorithm processed ECG and respiratory effort signals for the classification task and applied behavioral measurements (obtained from accelerometer and press button data) for the automatic adaptation task. When trained as a subject-independent classifier algorithm, the SleePic device was only able to correctly classify 74.94 ± 6.76% of the human rated sleep/wake data. By using the automatic adaptation method, the mean classification accuracy was improved to 92.98 ± 3.19%. A subject-independent classifier based on activity data only showed a comparable accuracy of 90.44 ± 3.57%.

Almazaydeh et al. [42] focused on an automated classification algorithm, which processed short duration epochs of the ECG data. The classification technique was based on SVM and had been trained and tested on sleep apnea recordings from subjects with and without OSA. The results showed that an automated classification system could recognize epochs of sleep disorders with a high accuracy of 96.5% or higher. Brignol et al. [43] proposed a phase space-based algorithm for automatic classification of sleep-wake states in humans using EEG data gathered over relatively short-time periods. The effectiveness of this approach was demonstrated through a series of experiments involving EEG data from seven healthy adult female subjects and was tested on epoch lengths ranging from 3 to 30-s. The performance of the phase space approach was compared to a two-dimensional state space approach using spectral power in two selected human-specific frequency bands. These powers were calculated by dividing integrated spectral amplitudes at selected human-specific frequency bands. The comparison demonstrated that the phase space approach gave better performance for the case of short as well as standard 30-s epoch lengths.

Majdi Bsoul et al. [44] developed a low-cost, real-time sleep apnea monitoring system called ‘Apnea MedAssist’ which was used for recognizing obstructive sleep apnea episodes with a high degree of accuracy for both home and clinical care applications. The fully automated system uses patient's single channel nocturnal ECG to extract feature sets and uses the support vector classifier (SVC) to detect apnea episodes. ‘Apnea MedAssist’ uses either the general adult subject-independent SVC model or subject-dependent SVC model and achieves a classification F-measure of 90% and a sensitivity of 96% for the subject-independent SVC. A two-stage procedure based on artificial neural networks for the automatic recognition of sleep spindles in a multi-channel electroencephalographic signal was introduced in [45]. Two different networks, i.e., a backpropagation multilayer perceptron and radial basis SVM, were proposed as the post-classifier and compared in terms of their classification performances. Visual evaluation, by two electroencephalographers (EEGers), of 19 channel EEG records of six subjects showed that the best performance was obtained with a radial basis SVM providing an average sensitivity of 94.6% and an average false detection rate of 4.0%.

## Database acquisition and preprocessing

**Figure 2:**
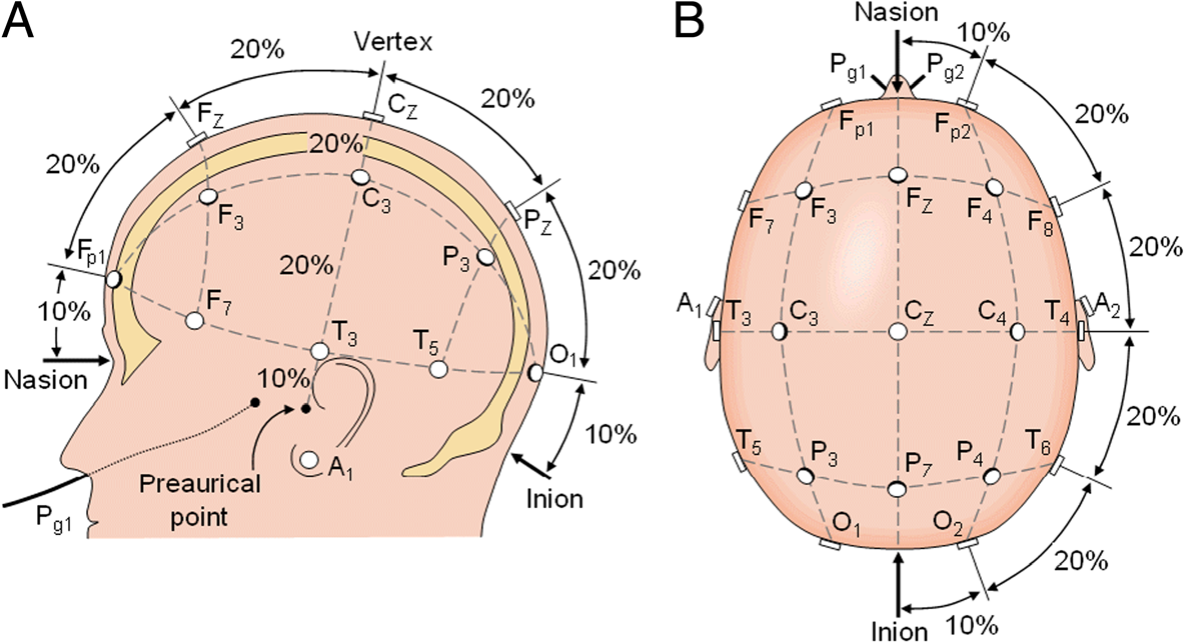
**The international 10-20 system seen from (A) left and (B) above the head.** A, ear lobe; C, central; Pg, nasopharyngeal; P, parietal; F, frontal; Fp, frontal polar; O, occipital. Source http://www.bem.fi/book/13/13.htm.

After ethics approval was obtained from the University of Toronto Research Ethics Office, subjects having no history of hearing loss or neurological problems were recruited by the research team. Written informed consent was obtained from all the subjects, and they were rewarded $100 for their participation. The stimulus, a wideband (700 to 3,000 Hz) chirp with 40.68 Hz rate, generated by Vivosonic Inc. Integrity™ V500 (Vivisonic Inc., Toronto, ON, Canada) was presented binaurally to both right and left ears using a Vivosonic Inc. ER-3A-ABR (Etymotic Research Inc., Elk Grove Village, IL, USA) insert earphone, loud enough to generate an ASSR but not too loud to cause discomfort to the participants. The stimulus has peaks at 60 dB HL and an equivalent sound pressure level and central frequency of 500 Hz with a sampling rate of 34.8 kHz. The device records EEG signals from 11 scalp sites of the international 10-20 system (Figure 2).

For our study, we used electrode sites F_z_, C_z_, C_3,4_, T_3,4_, A_1,2_, and O_z_. A reference electrode was used as the common electrode of all channels. A ground electrode was used to reduce the environmental noise, and a L_o_C was used for recording eye movements, in order to make sleep scoring based on raw EEG signals easier. An electrode cap by Bio-Medical Instruments Inc. (Warren, MI, USA) was custom designed with ten recording electrodes and two leads for the ear clip electrodes. A pair of 3½ inch DIN style EEG silver ear clips was used. A 10 mm in diameter gold cup electrode was used for recording eye movements. We used the Nicolet™ EEGwireless 32 (Natus Medical Incorporated, Pleasanton, CA, USA) amplifier to record the EEG amplification. Two extra electrodes were connected from the Integrity™ device stimuli generator to channel 25 of the EEG amplifier for recording the stimuli together with other EEG channels. The sampling frequency of the amplifier was *f*_s_ = 12 kHz, and all electrode impedances were below 5 kΩ.

**Figure 3:**
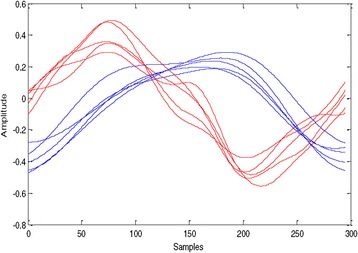
**40-Hz ASSR sweeps of wakefulness W**_**0**_ **state for subjects C (blue) and D (red) for channel Fz-A1A2.**

**Figure 4:**
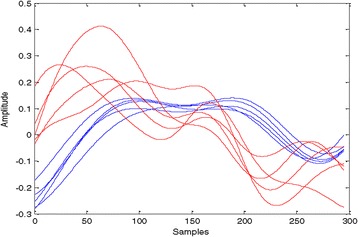
**40-Hz ASSR sweeps of deep sleep N**_**3**_ **state for subjects C (blue) and D (red) for channel Fz-A1A2.**

After recording, the raw mixed ASSR and EEG signals were reviewed and scored with conventional sleep scoring methods to awake *W*_*0*_ and three stages of sleep namely *N*_*1*_, *N*_*2*_, and *N*_*3*_. Signals of the *W*_*0*_ and *N*_*3*_ stages were transferred to MATLAB for preprocessing. Frequencies below 20 Hz and above 100 Hz were filtered out with third-order Butterworth low-pass and high-pass filters; the signals from seven recorded channels were synchronized and segmented into 295 sample sweeps. The EEG amplifier has 12 kHz sampling frequency but the Integrity stimulus was sampled with a 38.4 kHz sampling rate. Hence, the cycles for the 40-Hz response were not whole numbers. We got around this by only including cycles with 295 samples. This resulted in throwing out some of the data. However, this did not pose an issue as the required time to acquire data was not essential to the task. In almost all cases in the literature, ensemble averaging is used for extracting a 40-Hz ASSR signal from background noise [23,24]. Assuming the recorded signal as:

1 $$ {x}_i\left[n\right]={s}_i\left[n\right]+{r}_i\left[n\right] $$

where *x*_*i*_[*n*] is the ASSR in response to the *i* ^th^ sweep of the stimuli and *r*_*i*_[*n*] is the EEG and noise from other sources. Under the assumption that *s*_*i*_[*n*] is phase locked to the stimuli, noise *r*_*i*_[*n*] is zero mean, *E*(*r*_*i*_[*n*]) = 0, has constant variance, var(*r*_*i*_) = *σ* ^2^ and is uncorrelated from one sweep to another, *E*(*r*_*i*_[*n*]*r*_*j*_[*n* − *k*]) = *ρ*_*r*_[*k*]*δ*(*i* − *j*) ensemble average is an unbiased estimator and increases the variance of the noise. We used weighted ensemble averaging to extract the ASSR signals. The weights were calculated according to the Kalman filter coefficients. Each 40-Hz ASSR signal is extracted by averaging over a window of 900 sweeps. Each two adjacent windows have 83% overlap. After extracting 40-Hz ASSR signals, different features in time and frequency domain were compared in *W*_*0*_ and *N*_*3*_ stages in all seven channels. It is observed that peak-to-peak amplitude of 40-Hz ASSR decreases from *W*_*0*_ to *N*_*3*_. Figures 3 and 4 show five sweeps of ASSR during *W*_*0*_ and *N*_*3*_ for two subjects.

## Overview of algorithm

### LVQ classifier

The LVQ is a supervised classifier that was first studied by Kohonen [46]. To classify an input vector, it must be compared with all prototypes. The Euclidean distance metric is used to select the closest vector to the input vector. The input vector is classified to the same class as the nearest prototype.

**Figure 5:**
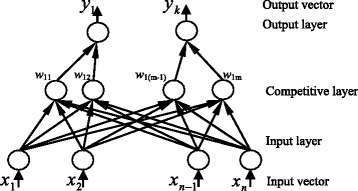
**Architecture of the LVQ classifier.**

The LVQ classifier (Figure 5) consists of an input layer, a hidden competitive layer, which learns to classify input vectors into subclasses and an output layer which transforms the competitive layer's classes into target classifications defined by the user. Only the winning neuron of the hidden layer has an output of one and other neurons have outputs of zero. The weight vectors of the hidden layer neurons are the prototypes, the number of which is usually fixed before training begins. The number of hidden neurons depends upon the complexity of the input-output relationship and significantly affects the results of classifier testing. Selection of the number of hidden neurons must be carefully made as it highly depends on the encompassed variability in the input patterns. Extensive experiments are performed to conduct the suitable number.

For a training set containing *n* input ensemble averaged sweeps of various subjects, each of these sweeps is labeled as being one of *k* classes which, in our case is 2, i.e., wakefulness and deep sleep states. The learning phase starts by initiating the weight vectors of neurons in the hidden layer. Then, the input vectors are presented randomly to the network. For each input vector *X*_*j*_, a winner neuron *W*_*i*_ is chosen to adjust its weight vector:

2 $$ \left\Vert {X}_j-{W}_i\right\Vert \le \left\Vert {X}_j-{W}_k\right\Vert, \mathrm{f}\mathrm{o}\mathrm{r}\;\mathrm{all}\;k\ne i $$

The weight vector *W*_*i*_(*t*) is updated to the next step *t* + 1 as follows:

3 $$ {W}_i\left(t+1\right)={W}_i(t)+\alpha \left({X}_j-{W}_i(t)\right) $$

if *X*_*j*_ and *W*_*i*_ belong to the same class

4 $$ {W}_i\left(t+1\right)={W}_i(t)-\alpha \left({X}_j-{W}_i(t)\right) $$

if *X*_*j*_ and *W*_*i*_ belong to different classes where 0 ≤ *α* ≤1 is the learning rate, which may be kept constant during training or may be decreasing monotonically with time for better convergence [46]. Otherwise, the weights remain the same. The training algorithm is stopped after reaching a pre-specified error limit. During the test phase, the distance of an input vector to each processing element of the hidden layer is computed and again the nearest element is declared as the winner. This, in turn, fires one output neuron, signifying a particular class.

### Efficient LVQ models

Careful selection of a feature extraction method highly simplifies the design of the classifier subsystem. Extraction of appropriate features is one of the most important tasks for a classification system. As it is impractical to match a given input signal with all the signal templates stored in the system, it is necessary to find a compact set of features that can represent as much of the useful information present in the original data as possible. Selection of *good* features is a crucial step in the process since the next stage sees only these features and acts upon them [47].

A generic LVQ-NN consists of three layers. The first layer is the input layer, which consists of as many neurons as the number of input samples of the signal to be classified. The hidden layer size is problem dependent. The number of hidden layer neurons (HN) should be suitable to capture the knowledge of the problem domain. For example, when training a neural network to recognize signals which belong to a number of classes (NC), then NC hidden layer neurons are required. To capture a large range of input pattern variability, a large number of hidden layer neurons is necessary. But, the problem is calculating how large should be this required number of hidden layer neurons.

**Figure 6:**
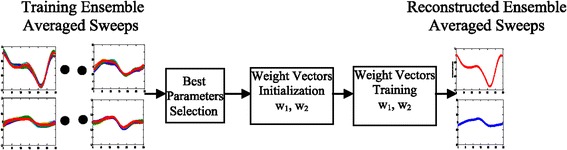
**LVQ algorithm flow for classification of wakefulness and sleep ensemble averaged sweeps.**

Visualizing the learned pattern of the hidden layer neurons, it is found that there are neurons with completely blurred patterns. These neurons are labeled blind neurons [48], as they do not see the signals that are clamped to the neurons of the input layer. Eliminating the blind neurons enhances the classifier performance, which is important for many biomedical applications. A classification model which considers reliability in the development of the model is very useful [40]. The compact LVQ network training algorithm for the classification system is illustrated in Figure 6. The algorithm based on efficient LVQ model parameters is as follows:

1. Select the network parameters:
  ✓ Input layer size = Ensemble averaged sweep signal size (295 neurons)
  ✓ Training set size = S (7 subjects) × 1,000 ensemble averaged sweeps
  ✓ Number of classes (NC) = 2 (wakefulness = *W*_*0*_ and sleep = *N*_*3*_)
  ✓ Hidden layer neurons; 2(min) ≤ HN ≤ 28(max)
  ✓ Learning rate (*α*) = 0.1
  ✓ Set up the target vector which specifies the target class of each pattern in the training set
  ✓ Display update rate = 100
  ✓ Arrange the input patterns of the training set as one-dimensional columns in an array (P)
  ✓ Number of training epochs (EP) = 2,500
2. Initialize an LVQ classifier: initialization of the weight matrix for competitive layer *w*_1_ and linear layer *w*_2_.
3. Start training of an LVQ classifier based on selected efficient model parameters.
4. Test the trained classifier on test sets and compute percentage of correct classification (pcc).
5. Get the best accuracy classification rate of wakefulness *W*_*0*_ and deep sleep *N*_*3*_.
6. Exit.

### Fusion at the features level

Multimodal classification systems help in achieving improved performance that may not be otherwise possible using a single classification system. However, an effective fusion scheme is necessary to combine the information presented by multiple domain experts. Pieces of evidence in a multi-classification system can be combined in many ways/levels and are generally divided into two categories [49], which we discuss below.

1. Before matching fusion. Fusion in this category integrates pieces of evidence before matching. This category fuses the information of multi-classification into the following levels.
  - Sensor level. At this level, the digital input signal is the result of sensing the same characteristic with two or more sensors or electrodes. The raw data acquired from multiple sensors can be processed and integrated to generate new data from which features can be extracted. For example, in the case of face biometrics, both 2-D texture information and 3-D depth (range) information (obtained using two different sensors) may be fused to generate a 3-D texture image of the face which could then be subjected to feature extraction and matching [50]. The combination of the input signals can provide noise cancelation, blind source separation [51], etc.
  - Feature level. The feature sets extracted from multiple data sources can be fused to create a new feature set to denote the identity. The geometric features of the hand, for example, may be augmented with the eigen coefficients of the face in order to construct a new high-dimensional feature vector [52]. A feature selection/transformation procedure may be adopted to produce a minimal feature set from the high-dimensional feature vector [53].
2. After matching fusion. Fusion in this category integrates pieces of evidence after matching. This includes the following levels.
  - Match score level. In this case, multiple classifiers output a set of match scores which are fused to generate a single scalar score [54]. As an example, the match scores generated by the face and hand modalities of a user may be combined via the simple sum rule in order to obtain a new match score which is then used to make the final decision [55].
  - Rank level. This type of fusion is relevant in identification systems where each classifier associates a rank with every enrolled identity (a higher rank indicating a good match). Thus, fusion entails consolidating the multiple ranks associated with an identity and determining a new rank that would aid in establishing the final decision.
  - Decision level*.* When each matcher outputs its own class label (i.e., accept or reject in a verification system, or the identity of a user in an identification system), a single class label can be obtained by employing techniques such as majority voting or behavior knowledge space [56]. In this last case, the Borda count method [57] can be used for combining the classifiers' outputs.

Integration at the feature level should provide better classification results than other levels of integration. This is because the features contain richer information about the input data than the matching score or the output decision of a classifier. However, integration at the feature level is difficult to achieve in practice due to the unknown relationship between the feature spaces of different classification systems, the concatenated feature vector with a very large dimensionality, the inaccessibility of the feature vectors of most commercial systems, and the computational cost to process the resultant vector.

In contrast, features-level fusion is easier to apply when the original characteristics are homogeneous. In this scenario, the single resultant feature vector needs to be calculated. We have adopted fusion at the features level to combine ASSR ensemble averaged sweeps of two electrode/channel vectors with the same dimensionality to concatenate into one vector which will also have the same dimensionality as the original vectors.

**Figure 7:**
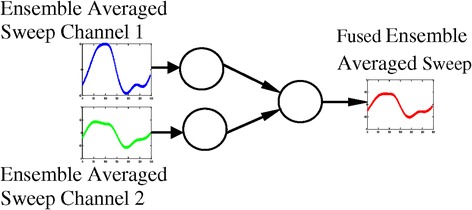
**Features-level fusion algorithm flow.**

A fused signal is one that is created by concatenating two ASSR ensemble averaged sweeps from two channels/electrodes of one subject. Figure 7 shows a features-level fusion algorithm flow using the compact LVQ-NN algorithm discussed above. This approach is particularly suitable for this type of signal processing because the NN is able to assimilate features of both ASSR ensemble averaged sweeps during its training phase. This features combining mechanism is inherent in the algorithm of the designated LVQ network (295 input neurons, 1 hidden neuron, 1 output neuron, 0.01 learning rate, and 700 training epochs).

Moreover, combining of features depends on a small number of parameters. This offers the advantage that the parameters of the combining algorithm are very easy to set out, so as to produce a fused ASSR ensemble averaged sweep signal that is different enough from the original sweeps to be a new one, as well as to avoid the creation of an over noisy signal. The target for training the LVQ network on both ensemble averaged sweeps from two electrodes is set to be same, which forces the network to join the features of both signals. The resulting fused signal indicates that when one hidden layer neuron responds to two ensemble averaged sweeps, it generates a mix of these signals.

### Hidden layer

Efficiency deals with the complexity of a learning machine in both space and time. The learning time must scale nicely with respect to the size of data sets. Since the size of the learning machine determines the memory required for implementation, a learning machine with a compact structure is preferred. Developing an adaptive learning system with a compact structure to achieve good performance is a challenging problem.

Experimental results [48] demonstrate that after convergence, most of the hidden layer neurons are redundant and do not evolve significantly and thus do not capture any data clusters. Typically, these neurons are initialized to points in the weight space that have relatively low overlap with the training data points. They play a little role in the pattern classification process, hence, may be eliminated without having significant effect on the detection accuracy rate.

Reducing the number of hidden layer neurons of NN to the product of subjects and classes, i.e., S × NC can help in increasing efficiency and performance of the whole system as many units not evolved properly during the training phase create confusion in the decision-making process. Thus, both the training time and the classification time are minimized.

### SVM classifier

The SVM [58] is a supervised learning algorithm useful for recognizing subtle patterns in complex datasets. The algorithm performs discriminative classification, learning by example to predict the classifications of previously unseen data. The algorithm has been applied in domains as diverse as text categorization, image recognition, and hand-written digit recognition. We have used the algorithm presented in [59] available online [60].

## Empirical results and discussion

The sleep patterns classification system is tested on 40-Hz ASSR ensemble averaged sweep signals recorded from eight human subjects [25]. A *N*_*3*_ or *SWS* was chosen due to its similarities to the signals generated during the surgical level of anesthesia [61]. In *SWS*, sensitivity to pain is the lowest relative to other sleep stages and arousal needs stronger stimuli. *SWS* is the switching of thalamus from tonic mode in which somatosensory information is transmitted through the thalamus, to its bursting mode, in which somatosensory information are inhibited from transmitting [61].

The performance of a classification system is expressed by parameters that relate to decision accuracy. A decision made by a system is labeled as either a *true* decision or a *false* decision [62]. For each type of decision, there are two possibilities, correct and incorrect. Hence, there are a total of four possible outcomes: a true state (*N*_*3*_ or *W*_*0*_) is correctly classified, a true state is incorrectly classified, a false state is correctly classified, and a false state is incorrectly classified. The decisions 1 and 3 are correct while 2 and 4 are incorrect. The confidence associated with different decisions may be characterized by the true distribution and the false distribution of classifications and used to establish the following two error rates [49]:

- False accept rate (FAR). The probability that the system incorrectly matches the input pattern to a non-matching template in the database. It measures the percent of invalid inputs which are incorrectly accepted.
- False reject rate (FRR). The probability that the system fails to detect a match between the input pattern and a matching template in the database. It measures the percent of valid inputs which are incorrectly rejected.

The FAR and the FRR are dual of each other. A smaller FRR usually leads to a larger FAR, while a smaller FAR usually implies a larger FRR. Normally, the system performance requirement is specified in terms of FAR. The performance of a biometric system may also be expressed using equal error rate (EER). EER is defined as the rate at which both accept and reject errors are equal. In general, the device with the lowest EER is considered to be the most accurate device for classifying biometric signals.

Various experiments are performed to explore the best parameters for a sleep pattern classification system using a developed LVQ fusion scheme and SVM classifier. First of all, a set of 100 ASSR ensemble averaged sweeps (50 *W*_*0*_ + 50 *N*_*3*_) of one subject was trained and a new set of 100 ensemble averaged sweeps (50 *W*_*0*_ + 50 *N*_*3*_) of the same subject was tested until zero error rate classification accuracy was achieved. This was repeated for all seven channel/electrode signals without any misclassification. Again, the experiments were carried out with a training of a bigger set of 500 ASSR ensemble averaged sweeps of five subjects, 5 × (50 *W*_*0*_ + 50 *N*_*3*_). Another set of 500 ensemble averaged sweeps 5 × (50 *W*_*0*_ + 50 *N*_*3*_) of the same subjects were tested until a zero for LVQ and very small for SVM error rate classification accuracy was secured for Fz-A1A2 channel (Tables 1 and 2).

**Table 1:** **LVQ single classifier error rate for ASSR ensemble averaged sweeps of same training and test subjects**

**Subject(s)**	**Training sweeps**	**Test sweeps**	**Error**
A	100(50 W_0_ + 50 N_3_)	100(50 W_0_ + 50 N_3_)	0%
B	100(50 W_0_ + 50 N_3_)	100(50 W_0_ + 50 N_3_)	0%
C	100(50 W_0_ + 50 N_3_)	100(50 W_0_ + 50 N_3_)	0%
D	100(50 W_0_ + 50 N_3_)	100(50 W_0_ + 50 N_3_)	0%
E	100(50 W_0_ + 50 N_3_)	100(50 W_0_ + 50 N_3_)	0%
F	100(50 W_0_ + 50 N_3_)	100(50 W_0_ + 50 N_3_)	0%
G	100(50 W_0_ + 50 N_3_)	100(50 W_0_ + 50 N_3_)	0%
H	100(50 W_0_ + 50 N_3_)	100(50 W_0_ + 50 N_3_)	0%
A, B, C, D, E	500(50 W_0_ + 50 N_3_) × 5	500(50 W_0_ + 50 N_3_) × 5	0%

For channel Fz-A1A2.

**Table 2:** **SVM single classifier error rate for ASSR ensemble averaged sweeps of same training and test subjects**

**Subject(s)**	**Training sweeps**	**Test sweeps**	**Error**
A	100(50 W_0_ + 50 N_3_)	100(50 W_0_ + 50 N_3_)	1%
B	100(50 W_0_ + 50 N_3_)	100(50 W_0_ + 50 N_3_)	1%
C	100(50 W_0_ + 50 N_3_)	100(50 W_0_ + 50 N_3_)	1%
D	100(50 W_0_ + 50 N_3_)	100(50 W_0_ + 50 N_3_)	2%
E	100(50 W_0_ + 50 N_3_)	100(50 W_0_ + 50 N_3_)	0%
F	100(50 W_0_ + 50 N_3_)	100(50 W_0_ + 50 N_3_)	2%
G	100(50 W_0_ + 50 N_3_)	100(50 W_0_ + 50 N_3_)	0%
H	100(50 W_0_ + 50 N_3_)	100(50 W_0_ + 50 N_3_)	1%
A, B, C, D, E	500(50 W_0_ + 50 N_3_) × 5	500(50 W_0_ + 50 N_3_) × 5	3%

For channel Fz-A1A2.

The next phase of experiments involved training the NN classifier with ASSR ensemble averaged sweeps of five subjects and testing the ensemble averaged sweeps of the sixth subject, which was not part of the training. The sleep patterns classification system faced a real challenge in classifying the ensemble averaged sweeps of a subject, which did not form the basis of system training. Sleep patterns classification systems that rely on physiological signals suffer from inter-subject differences that make accurate classification within a single, subject-independent model difficult [41]. Although, the sleep and/or wakefulness patterns for different subjects vary slightly in shape and in amplitude levels [24], the devised LVQ NN system is still capable of classifying the ASSR ensemble averaged sweeps with no error rate. However, SVM classification error rate jumps to significant value (Tables 3 and 4).

**Table 3:** **LVQ single classifier error rate for ASSR ensemble averaged sweeps of different training and test subjects**

**Training subjects**	**Test subject**	**Training sweeps**	**Test sweeps**	**Error**
A, B, C, D, E	F	500(50 W_0_ + 50 N_3_) × 5	100(50 W_0_ + 50 N_3_)	0%
A, B, C, D, F	E	500(50 W_0_ + 50 N_3_) × 5	100(50 W_0_ + 50 N_3_)	0%
A, B, C, E, F	D	500(50 W_0_ + 50 N_3_) × 5	100(50 W_0_ + 50 N_3_)	0%
A, B, D, E, F	C	500(50 W_0_ + 50 N_3_) × 5	100(50 W_0_ + 50 N_3_)	0%
A, C, D, E, F	B	500(50 W_0_ + 50 N_3_) × 5	100(50 W_0_ + 50 N_3_)	0%
B, C, D, E, F	A	500(50 W_0_ + 50 N_3_) × 5	100(50 W_0_ + 50 N_3_)	0%

For channel Fz-A1A2.

**Table 4:** **SVM single classifier error rate for ASSR ensemble averaged sweeps**

**Training subjects**	**Test subject**	**Training sweeps**	**Test sweeps**	**Error**
A, B, C, D, E	F	500(50 W_0_ + 50 N_3_) × 5	100(50 W_0_ + 50 N_3_)	9%
A, B, C, D, F	E	500(50 W_0_ + 50 N_3_) × 5	100(50 W_0_ + 50 N_3_)	28%
A, B, C, E, F	D	500(50 W_0_ + 50 N_3_) × 5	100(50 W_0_ + 50 N_3_)	13%
A, B, D, E, F	C	500(50 W_0_ + 50 N_3_) × 5	100(50 W_0_ + 50 N_3_)	14%
A, C, D, E, F	B	500(50 W_0_ + 50 N_3_) × 5	100(50 W_0_ + 50 N_3_)	20%
B, C, D, E, F	A	500(50 W_0_ + 50 N_3_) × 5	100(50 W_0_ + 50 N_3_)	23%

Of different training and test subjects for channel Fz-A1A2.

The reliability of the results depends heavily on the accuracy of statistical parameters involved in classifiers in general. The obtained results cannot be accurately estimated with only a small number of training samples. Therefore, it is of vital importance to include the minimum number of training samples and to ensure that the derived conclusions have a good degree of consistency. To increase the reliability of our estimations, a similar set of experiments was repeated with a higher number of subjects and/or ASSR ensemble averaged sweeps (Tables 5 and 6).

**Table 5:** **LVQ single classifier error rate for large set of ASSR ensemble averaged sweeps**

**Training subjects**	**Test subject**	**Training sweeps**	**Test sweeps**	**Error**
A, B, C, D, E, F, G	H	7,000(500 W_0_ + 500 N_3_) × 7	1,000(500 W_0_ + 500 N_3_)	7%
A, B, C, D, E, F, H	G	7,000(500 W_0_ + 500 N_3_) × 7	1,000(500 W_0_ + 500 N_3_)	0%
A, B, C, D, E, G, H	F	7,000(500 W_0_ + 500 N_3_) × 7	1,000(500 W_0_ + 500 N_3_)	0%
A, B, C, D, F, G, H	E	7,000(500 W_0_ + 500 N_3_) × 7	1,000(500 W_0_ + 500 N_3_)	6.6%
A, B, C, E, F, G, H	D	7,000(500 W_0_ + 500 N_3_) × 7	1,000(500 W_0_ + 500 N_3_)	0%
A, B, D, E, F, G, H	C	7,000(500 W_0_ + 500 N_3_) × 7	1,000(500 W_0_ + 500 N_3_)	0%
A, C, D, E, F, G, H	B	7,000(500 W_0_ + 500 N_3_) × 7	1,000(500 W_0_ + 500 N_3_)	0%
B, C, D, E, F, G, H	A	7,000(500 W_0_ + 500 N_3_) × 7	1,000(500 W_0_ + 500 N_3_)	5.5%

Of different training and test subjects for channel Fz-A1A2.

**Table 6:** **SVM single classifier error rate for large set of ASSR ensemble averaged sweeps**

**Training subjects**	**Test subject**	**Training sweeps**	**Test sweeps**	**Error**
A, B, C, D, E, F, G	H	7,000(500 W_0_ + 500 N_3_) × 7	1,000(500 W_0_ + 500 N_3_)	32%
A, B, C, D, E, F, H	G	7,000(500 W_0_ + 500 N_3_) × 7	1,000(500 W_0_ + 500 N_3_)	16%
A, B, C, D, E, G, H	F	7,000(500 W_0_ + 500 N_3_) × 7	1,000(500 W_0_ + 500 N_3_)	13%
A, B, C, D, F, G, H	E	7,000(500 W_0_ + 500 N_3_) × 7	1,000(500 W_0_ + 500 N_3_)	31%
A, B, C, E, F, G, H	D	7,000(500 W_0_ + 500 N_3_) × 7	1,000(500 W_0_ + 500 N_3_)	16%
A, B, D, E, F, G, H	C	7,000(500 W_0_ + 500 N_3_) × 7	1,000(500 W_0_ + 500 N_3_)	17%
A, C, D, E, F, G, H	B	7,000(500 W_0_ + 500 N_3_) × 7	1,000(500 W_0_ + 500 N_3_)	22%
B, C, D, E, F, G, H	A	7,000(500 W_0_ + 500 N_3_) × 7	1,000(500 W_0_ + 500 N_3_)	29%

Of different training and test subjects for channel Fz-A1A2.

It is difficult to predict which channels/electrodes will produce noisy input data and unacceptable error rates that will challenge the performance of classification systems. These random degradations make it difficult to classify the wakefulness or deep sleep state and lower the performance of classification algorithms. Hence, in general, more channels/electrodes are used to record/monitor the sleep patterns to compensate for unexpected errors.

The final phase of experiments was carried out with input vectors of the NN sleep patterns classification system. The resultant ASSR ensemble averaged sweep signals from two channels/electrodes of one subject obtained at the features level. Tables 7 and 8 show various results of LVQ and SVM classifiers with fused ASSR ensemble averaged sweeps.

**Table 7:** **LVQ multimodal classifier error rate for large set of ASSR ensemble averaged sweeps**

**Training subjects**	**Test subject**	**Training sweeps**	**Test sweeps**	**Error**
A, B, C, D, E, F, G	H	7,000(500 W_0_ + 500 N_3_) × 7	1,000(500 W_0_ + 500 N_3_)	17.2%
A, B, C, D, E, F, H	G	7,000(500 W_0_ + 500 N_3_) × 7	1,000(500 W_0_ + 500 N_3_)	0%
A, B, C, D, E, G, H	F	7,000(500 W_0_ + 500 N_3_) × 7	1,000(500 W_0_ + 500 N_3_)	0%
A, B, C, D, F, G, H	E	7,000(500 W_0_ + 500 N_3_) × 7	1,000(500 W_0_ + 500 N_3_)	7.9%
A, B, C, E, F, G, H	D	7,000(500 W_0_ + 500 N_3_) × 7	1,000(500 W_0_ + 500 N_3_)	0%
A, B, D, E, F, G, H	C	7,000(500 W_0_ + 500 N_3_) × 7	1,000(500 W_0_ + 500 N_3_)	0%
A, C, D, E, F, G, H	B	7,000(500 W_0_ + 500 N_3_) × 7	1,000(500 W_0_ + 500 N_3_)	0%
B, C, D, E, F, G, H	A	7,000(500 W_0_ + 500 N_3_) × 7	1,000(500 W_0_ + 500 N_3_)	17.9%

Of different training and test subjects for channels C4-A1A2 and Fz-A1A2.

**Table 8:** **SVM multimodal classifier error rate for large set of ASSR ensemble averaged sweeps**

**Training subjects**	**Test subject**	**Training sweeps**	**Test sweeps**	**Error**
A, B, C, D, E, F, G	H	7,000(500 W_0_ + 500 N_3_) × 7	1,000(500 W_0_ + 500 N_3_)	30%
A, B, C, D, E, F, H	G	7,000(500 W_0_ + 500 N_3_) × 7	1,000(500 W_0_ + 500 N_3_)	23%
A, B, C, D, E, G, H	F	7,000(500 W_0_ + 500 N_3_) × 7	1,000(500 W_0_ + 500 N_3_)	19%
A, B, C, D, F, G, H	E	7,000(500 W_0_ + 500 N_3_) × 7	1,000(500 W_0_ + 500 N_3_)	23%
A, B, C, E, F, G, H	D	7,000(500 W_0_ + 500 N_3_) × 7	1,000(500 W_0_ + 500 N_3_)	14%
A, B, D, E, F, G, H	C	7,000(500 W_0_ + 500 N_3_) × 7	1,000(500 W_0_ + 500 N_3_)	20%
A, C, D, E, F, G, H	B	7,000(500 W_0_ + 500 N_3_) × 7	1,000(500 W_0_ + 500 N_3_)	16%
B, C, D, E, F, G, H	A	7,000(500 W_0_ + 500 N_3_) × 7	1,000(500 W_0_ + 500 N_3_)	24%

Of different training and test subjects for channels C4-A1A2 and Fz-A1A2.

The efficiency of the proposed LVQ architecture is evaluated on both the time and the space scale. By setting the number of HN equal to the product of subjects and classes (S × NC), the network memory requirements for the internal representation of target signals was condensed and the processing speed was enhanced. Specifically, both the training time of the network and the test time of the ASSR ensemble averaged sweep signals were reduced. This makes it feasible for large data training and test samples in real-time application domains as the storage requirements of the sleep pattern classification system with fusion scheme are the same as a single channel/electrode classification system.

## Conclusions

The manual scoring of sleep patterns (wakefulness *W*_*0*_ and deep sleep *N*_*3*_) is a time-consuming process, in which sleep states are normally determined using EEG signals of human subjects. This paper considered a LVQ-NN- and SVM-based automatic classification algorithm for 40 Hz ASSR ensemble averaged signals. 40 Hz ASSR signals were extracted by averaging over 900 sweeps on a 30-s window from EEG. EEG signals were recorded from eight human subjects. *N*_*3*_ deep sleep state was selected for this task because of its resemblance to states of consciousness and wakefulness achieved by the administration of general anesthesia given to patients during clinical surgery. Future studies can thus observe the depth of general anesthesia by classifying consciousness and wakefulness states of patients with 40-Hz ASSR.

A single classifier has the weakness of not providing the confidence level required in monitoring sleep patterns. As a result, a multimodal classifier using fusion scheme at the features level by combining signals from two electrodes/channels was used to enhance the classification confidence. Our three-fold objectives of a) generating an automatic classification of sleep patterns (wakefulness *W*_*0*_ and deep sleep *N*_*3*_) based on an adaptive LVQ-NN and SVM with 40-Hz ASSR input signals, b) developing a features-level fusion approach for combining a 40-Hz ASSR ensemble averaged sweep of signals generated from two separate electrodes/channels, and c) classifying sleep patterns with the resultant ASSR sweep signals to enhance the decision confidence level have been accomplished. LVQ-NN outperforms as compared to the SVM for 40-Hz ASSR ensemble averaged signals classification for observing sleep patterns.
